# 小细胞肺癌免疫治疗的临床研究进展

**DOI:** 10.3779/j.issn.1009-3419.2020.105.02

**Published:** 2020-11-20

**Authors:** 阳阳 许, 平 展, 勇 宋

**Affiliations:** 1 210002 南京，南京医科大学金陵临床医学院(东部战区总医院)呼吸和危重症医学科 Department of Respiratory and Critical Care Medicine, Jinling Hospital, Nanjing Medical University, Nanjing 210002, China; 2 210002 南京，东部战区总医院(南京大学医学院金陵医院)呼吸和危重症医学科 Department of Respiratory and Critical Care Medicine, Jinling Hospital, Nanjing University School of Medicine, Nanjing 210002, China

**Keywords:** 小细胞肺癌, 免疫治疗, 标志物, Small cell lung cancer, Immunotherapy, Biomarkers

## Abstract

小细胞肺癌(small cell lung cancer, SCLC)是一种“顽固性癌症”，以快速生长和早期广泛转移为特征，大约70%的患者在确诊时就已经处于广泛期。尽管对一线含铂双联化疗反应率高，但是几乎所有患者随后即不可避免地复发且对二线治疗的反应较差。由于SCLC具有高肿瘤突变负荷及免疫源性，这提示免疫治疗也许对其有效。在过去的几年中，一些临床试验评估了以细胞毒性T淋巴细胞相关抗原4(cytotoxic T lymphocyte-associated antigen-4, CTLA-4)和程序性死亡受体1(programmed death 1, PD-1)/程序性死亡配体-1(programmed death ligand-1, PD-L1)抑制剂为主的检查点抑制剂在SCLC患者中的治疗效果，并展示了良好的生存前景。本文总结了这些试验中免疫检查点抑制剂单独或联合应用于SCLC一线治疗、维持治疗和二线或以上治疗的结果，并对其中的预测因素进行了综述以确定其临床价值。

## Abstract (en)

Small cell lung cancer (SCLC) is a "refractory cancer" characterized by rapid growth and extensive early metastasis. About 70% of patients are already in the extensive stage at the time of diagnosis. Despite the high response rate to platinum-contained first-line chemotherapy, almost all patients subsequently experienced inevitable recurrence and had poor response to second-line treatment. The high mutation load and immunogenicity of SCLC suggest that immunotherapy may be effective for SCLC patients. Over the past few years, several clinical trials have evaluated the efficacy of checkpoint inhibitors [mainly cytotoxic T lymphocyte-associated antigen-4 (CTLA-4) and programmed death 1 (PD-1)/programmed death ligand-1 (PD-L1) inhibitors] in SCLC patients and demonstrated promising survival prospects. This article reviewed the clinical studies of immune checkpoint inhibitors (ICIs) in the first-line, maintenance and second or more line treatment of SCLC. Besides, predictive biomarkers were discussed to select suitable patients for immunotherapy effectively.

## 1 背景及目前治疗状况

小细胞肺癌(small cell lung cancer, SCLC)占肺癌总数的10%-15%。大约2/3的患者在发现时已经有转移性疾病，中位总生存期(overall survival, OS)只有8个月-13个月^[1, 2]^。目前SCLC的一线治疗方案仍然是铂-依托泊苷化疗(Etoposide+Cisplatin/Carboplatin, EP)，对于局限期小细胞肺癌(limited-stage SCLC, LS-SCLC)患者，则可以适当联用放疗^[3, 4]^。一般来说，该方案的初期应答率较好，广泛期(extensive-stage, ES)患者的反应率可达60%-80%。但是，大多数患者在初始治疗后不久就出现复发性疾病且病情相对较难控制，几乎没有有效的治疗选择，预后非常糟糕：LS-SCLC和ES-SCLC的5年总生存率分别为10%-20%和1%-2%^[5, 6]^。对于一线化疗后失败或复发的患者常首选拓扑替康治疗。然而，它的治疗效果有限，对铂类化疗敏感患者的应答率约为23%，铂耐药或难治性患者的应答率仅有9%，并且这些反应并不持久。此外，拓扑替康的毒性特征，尤其是血液学毒性，可能会限制其在许多患者中的使用^[7, 8]^。因此，迫切需要找到用于治疗这些患者的新型药物。在过去的几年中，有关SCLC的免疫治疗取得了新的进展，本文对相关的临床研究进行了回顾。

## 2 SCLC免疫治疗的基本原理

一方面，SCLC与烟草消费密切相关，大约98%的SCLC患者有吸烟史^[9]^，而有研究^[10]^证实，烟草可以通过60多种能够与DNA结合并使其发生突变的化学物质发挥致癌作用。因此，SCLC具有高肿瘤突变负担(tumor mutational burden, TMB)，该特征能够导致肿瘤新抗原的释放，从而引发针对肿瘤细胞的适应性免疫反应，这为SCLC的免疫治疗带来了希望^[11]^。

另一方面，约20%-40%的SCLC患者存在副肿瘤综合征，具有一定的免疫源性^[12]^。Maddison等^[13]^发现，患有Lambert-Eaton综合征的SCLC患者生存率明显提高。即使临床上没有诊断出明显的副肿瘤综合征，自身抗体如抗神经元核抗体阳性的患者也具有明显的生存优势，这一定程度上反映了SCLC诱发体液免疫应答的能力^[14]^。此外，既往回顾性研究^[15]^表明宿主免疫状态与SCLC的预后密切相关，癌症相关的炎症状态是肿瘤进展和生存的重要因素。与更好的预后相关的免疫学指标包括肿瘤浸润淋巴细胞、肿瘤内T细胞和细胞毒性CD8细胞的高发生率^[16]^。抑制性免疫功能表明SCLC的预后较差，肿瘤浸润中FOXP3+细胞比例、CD14(+)HLA-DR-/Low髓源性抑制细胞的数量和出现频率是SCLC预后较差的预测因子^[17, 18]^。这些临床证据进一步说明了免疫应答的重要性以及SCLC免疫治疗的可行性。

## 3 SCLC免疫治疗临床研究进展

### 3.1 一线治疗

目前有关免疫检查点抑制剂(immune checkpoint inhibitors, ICIs)单独应用于SCLC一线治疗的研究很少，这可能与SCLC进展迅速、不进行化疗潜在风险大有关。考虑到化疗与免疫治疗之间有着潜在的协同作用：细胞毒性化疗药物可以诱导免疫源性细胞死亡，产生分子信号，促进癌细胞死亡碎片被抗原呈递细胞摄取，协同ICIs提高抗肿瘤活性，干扰肿瘤细胞的生长和传播^[19, 20]^。因此大多数试验探索了化疗和免疫治疗联合应用的方法。

免疫联合化疗Ipilimumab是最早在SCLC中与化疗联用的细胞毒性T淋巴细胞相关抗原4(cytotoxic T lymphocyte-associated antigen-4, CTLA-4)抑制剂。如表 1所示，尽管CA184-041研究发现Ipilimumab联合化疗(紫杉醇+卡铂)可以使免疫相关无进展生存(progression free survival, PFS)提高^[21]^，但是在随后进行的Ⅲ期临床试验中，Ipilimumab联合铂-依托泊苷并未导致总生存的显著改善(HR=0.94; 95%CI: 0.81-1.09; *P*=0.377, 5)。这可能是因为在肿瘤微环境中缺乏相应的T细胞激活，Ipilimumab无法有效地在SCLC中产生足够强的抗肿瘤反应^[22]^。另一项评估Ipilimumab联合EP一线治疗ES-SCLC疗效的单臂试验也因为过量毒性而终止，1年无进展生存率仅为15.8%^[23]^。

**表 1:** 已经完成的免疫治疗应用于小细胞肺癌的临床试验 Completed clinical trials with immune checkpoint inhibitors for Small cell lung cancer (SCLC)

Trial	Author	Year	Phase	No.	Patient selection	Treatment regimen	Primary endpoint	Results	Toxicity
First-line treatment									
Immunotherapy combined with chemotherapy									
CA184-041	Reck	2013	II	42 43 45	Previously untreated ES-SCLC	Arm A (Phased): placebo/paclitaxel /carboplatin → ipilimumab (10 mg/kg)/paclitaxel/carboplatin; Arm B (Concurrent): ipilimumab (10 mg/kg)/paclitaxel/carboplatin → placebo/paclitaxel/carboplatin; Arm C (Control): placebo/paclitaxel/carboplatin	irPFS	Arm A *vs* Arm B *vs* Arm C mirPFS: 6.4 mo *vs* 5.7 mo *vs* 5.3 mo; mPFS: 5.2 mo *vs* 3.9 mo *vs* 5.2 mo; mOS: 12.5 mo *vs* 9.1 mo *vs* 10.5 mo	Grade 3/4: 53.0% *vs* 45.0% *vs* 43.0%
CA184-156	Reck	2016	III	478 476	Previously untreated ES-SCLC	Arm A: etoposide+platinum+ipilimumab (10 mg/kg); Arm B: etoposide+platinum+placebo	OS	mOS: 11.0 mo *vs* 10.9 mo; mPFS: 4.6 mo *vs* 4.4 mo; mDOR: 4.01 mo *vs* 3.45 mo	Grade 3/4: 48.0% *vs* 44.0%; Death: 1% *vs* < 1%
NCT01331525	Arriola	2016	II	38	Previously untreated ES-SCLC	Carboplatin+etoposide+ipilimumab (10 mg/kg)	1-year PFS	1-year PFS rate: 15.8%; mPFS: 6.9 mo; mirPFS: 7.3 mo; mOS: 17.0 mo; ORR: 72.4%	Grade 3/4: 89.7%; Death: 13%
IMpower133	Horn	2018	III	201 202	Previously untreated ES-SCLC	Arm A: carboplatin+etoposide+atezolizumab 1, 200 mg; Arm B: carboplatin+etoposide+placebo	PFS, OS	mOS: 12.3 mo *vs* 10.3 mo; mPFS: 5.2 mo *vs* 4.3 mo; mDOR: 4.2 mo *vs* 3.9 mo	Grade 3/4: 39.9% *vs* 24.5%; Death: 1.5% *vs* 1.5%
CASPIAN	Paz-Ares	2019	III	268 269	Previously untreated ES-SCLC	Arm A: durvalumab 1, 500 mg+carboplatin/cisplatin+etoposide; Arm B: carboplatin/cisplatin+etoposide	OS	mOS: 13 mo *vs* 10.3 mo; mPFS: 5.1 mo *vs* 5.4 mo	Grade 3/4: 62.0% *vs* 62.0%; Deaths: 5% *vs* 6%
Maintenance treatment									
Monotherapy									
NCT02359019	Gadgeel	2018	II	45	ES-SCLC with a response or stable disease after ChT	Pembrolizumab 200 mg	PFS	mPFS: 1.4 mo; 1-year PFS rate: 13%; mOS: 9.6 mo; 1-year OS rate: 37%	Grade 3/4: 5.0%; Deaths: 4.4%
Double checkpoint inhibitor combination									
CheckMate 451	Owonikoko	2019	III	279 280 275	ES-SCLC with a response or stable disease after ChT	Arm A: nivolumab 1 mg/kg+ipilimumab 3 mg/kg → nivolumab 240 mg; Arm B: nivolumab 240 mg; Arm C: placebo	OS for Arm A *vs* Arm C	OS: Arm A *vs* Arm C: HR=0.92; 95%CI: 0.75-1.12; Arm B *vs* Arm C: HR=0.84; 95%CI: 0.69-1.02. PFS: Arm A *vs* Arm C: HR=0.72; 5%CI: 0.60-0.87; Arm B *vs* Arm C: HR=0.67; 95%CI: 0.56-0.81	Grade 3/4: 52.0% *vs* 12.0% *vs* 8.0%; Deaths: 2.5% *vs* < 1% *vs* < 1%
Second-line or further treatments									
Monotherapy									
CheckMate 032	Antonia	2016	I/II, non-randomized cohort	98 61 54 3	Progressed after at least one platinum-based ChT, ES-SCLC/LS-SCLC	Arm A: nivolumab 3 mg/kg; Arm B: nivolumab 1 mg/kg+ ipilimumab 3 mg/kg; Arm C: nivolumab 3 mg/kg+ipilimumab 1 mg/kg; Arm D: nivolumab 1 mg/kg+ipilimumab 1 mg/kg	ORR	ORR: 10% *vs* 23% *vs* 19% *vs* 33%; mOS: 4.4 mo *vs* 7.7 mo *vs* 6 mo *vs* NA; mPFS: 1.4 mo *vs* 2.6 mo *vs* 1.4 mo *vs* NA	Grade 3/4: 13.0% *vs* 30.0% *vs* 19.0% *vs* 0; Deaths: 0 *vs* 3% *vs* 3% *vs* 0
CheckMate 032	Ready	2019	I/II, randomized cohort	147 96	Progressed after at least one platinum-based ChT, ES-SCLC/LS-SCLC	Arm A: nivolumab 3 mg/kg; Arm B: nivolumab 1 mg/kg+ ipilimumab 3 mg/kg	ORR	ORR: 11.6% *vs* 21.9%; mDOR: 15.8 mo *vs* 10.0 mo; mPFS: 1.4 mo *vs* 1.5 mo; mOS: 5.7 mo *vs* 4.7 mo	Grade 3/4: 12.9% *vs* 37.5%; Deaths: < 1% *vs* 3%
CheckMate 331	Reck	2018	III	284 285	Progressed after 1L platinum-based ChT, SCLC	Arm A: nivolumab; Arm B: topotecan/amrubicin	OS	mOS: 7.5 mo *vs* 8.4 mo; mPFS: 1.4 mo *vs* 3.8 mo; ORR: 14% *vs* 16%	Grade 3/4: 14.0% *vs* 73.0%; Deaths: < 1% *vs* 1%
KEYNOTE-028	Ott	2017	Ib	24	Progressed after platinum-based ChT, ES-SCLC, PD-L1: ≥1%	Pembrolizumab 10 mg/kg	Safety, ORR	ORR: 33.3%; mPFS: 1.9 mo; mOS: 9.7 mo; mDOR: 19.4 mo	Grade 3-5: 33.3%
KEYNOTE-158	Chung	2018	II	107	Progressed after platinum-based ChT, SCLC	Pembrolizumab 200 mg	ORR	ORR: 18.7%; mPFS: 2.0 mo; mOS: 9.1 mo	All grades: 59.0%; Deaths: < 1%
NCT01375842	Sequist	2016	Ia	17	Progressed after platinum-based ChT, ES-SCLC	Atezolizumab 15 mg/kg or 1, 200 mg	NA	irORR: 24%; mPFS: 1.5 mo; mOS: 5.9 mo	All grades: 65.0%
IFCT-1603	Pujol	2019	II, non-comparative	49 24	Progressed after platinum-based ChT, SCLC	Arm A: atezolizumab 1, 200 mg; Arm B: topotecan /re-induction of initial ChT	ORR at 6 weeks	ORR at 6 weeks: 2.3% *vs* 10%; DCR at 6 weeks: 20.9% *vs* 65%; mPFS: 1.4 mo *vs* 4.3 mo; mOS: 9.5 mo *vs* 8.7 mo	Grade 3/4: 4.2%
NCT01693562	Goldman	2018	I/II	21	Pretreated ES-SCLC	Durvalumab 10 mg/kg	Safety	ORR: 9.5%; mPFS: 1.5 mo; mOS: 4.8 mo	Grade 3/4: 0
Immunotherapy combined with chemotherapy									
NCT0255143	Kim	2019	II	26	Progressed after platinum-based ChT, ES-SCLC	Paclitaxel 175 mg/m2+pembrolizumab 200 mg	ORR	ORR: 23.1%; mPFS: 5.0 mo; mOS: 9.1 mo	Grade 3/4: 38.5%
Double checkpoint inhibitor combination									
NCT02261220	Cho	2018	I	30	Pretreated ES-SCLC	Durvalumab 20 mg/kg+tremelimumab 1 mg/kg	NA	ORR: 13.3%; mDOR: 18.9 mo; mPFS: 1.8 mo; mOS: 7.9 mo	Grade 3/4: 23.0%
BALTIC	Bondarenko	2018	II	21	Platinum-refractory/resistant ES-SCLC	Durvalumab 1, 500 mg+tremelimumab 75 mg → durvalumab 1, 500 mg	ORR	ORR: 9.5%	Grade 3/4: 48.0%
Immunotherapy combined with radiotherapy									
NCT02701400	Owonikoko	2019	II	8 9	Relapsed SCLC, received≤2L of therapy	Arm A: tremelimumab 1, 500 mg+durvalumab 75 mg; Arm B: tremelimumab 1, 500 mg+durvalumab 75 mg +SBRT	NA	mPFS: 2.1 mo *vs* 3.3 mo; mOS: 2.6 mo *vs* 5.7 mo	NA
irPFS: immune-related progression-free survival; No.: number of patients; ChT: chemotherapy; mo: month; OS: overall survival; PFS: progression free survival; mDOR: median duration of response; ORR: objective response rate; irORR: confirmed ORR by irRC; DCR: disease control rate; SBRT: stereotactic body radiotherapy; NA: not available; HR: hazard ratio; ≤2L: not more than 2 lines of therapy; 1L: first-line treatment; ES-SCLC: extensive-stage small cell lung cancer; LS-SCLC: limited-stage small cell lung cancer; PD-L1: programmed death ligand-1.									

尽管有关Ipilimumab研究的结果不尽如人意，但它确实证明了化疗联合免疫治疗应用于SCLC的可行性。IMpower133研究发现，EP联合Atezolizumab组的中位OS较单纯化疗组长2个月(*P*=0.007)。根据临床、病理和分子特征选择的所有预先定义的亚组[如性别、年龄、血液肿瘤突变负荷、美国东部肿瘤协作组(Eastern Cooperative Oncology Group, ECOG)评分、脑转移、肝转移等]在总生存和无进展生存方面的获益也是一致的^[24]^。可见，在卡铂和依托泊苷中添加Atezolizumab可显著延长SCLC患者的总生存。该联合治疗方案于2019年先后被美国国家综合癌症网络(National Comprehensive Cancer Network, NCCN)指南、美国食品药品监督管理局(Food and Drug Administration, FDA)批准作为ES-SCLC患者的一线治疗选择(图 1)。

**图 1:**
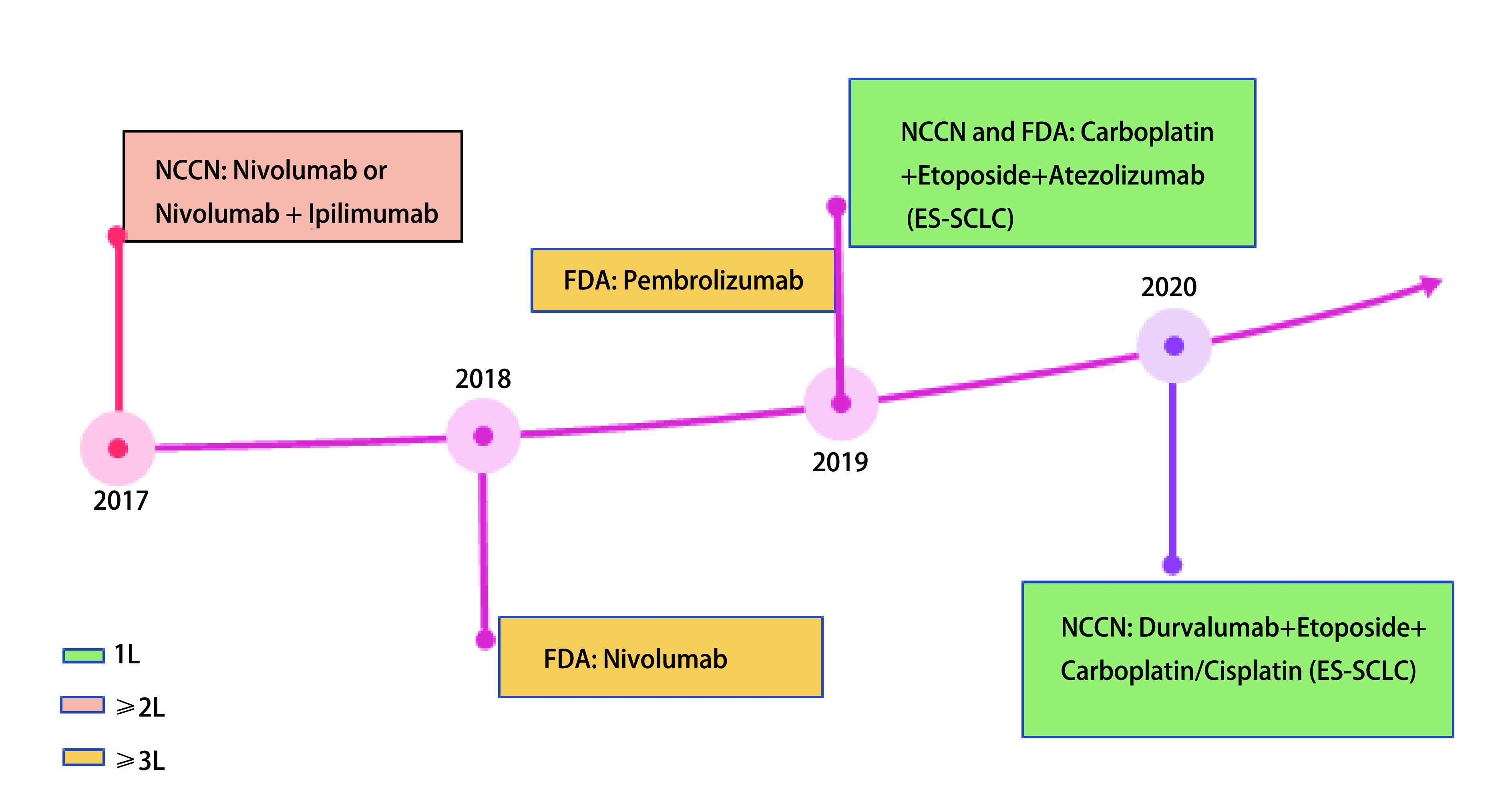
小细胞肺癌免疫治疗进展的时间轴 Timeline of immunotherapy progress for SCLC. 1L: first-line treatment; 2L: 2 lines of therapy; 3L: 3 lines of therapy.

CASPIAN研究(一项随机对照的Ⅲ期临床试验)探讨了Durvalumab联合铂-依托泊苷在ES-SCLC患者一线治疗中的疗效。最新的中期分析结果发现和单纯化疗组(*n*=269)相比，durvalumab联合铂-依托泊苷(*n*=268)可显著改善患者的总生存，其危险比为0.73(95%CI: 0.59-0.91; *P*=0.004, 7)。Durvalumab联合化疗的毒性也是可接受的，发生导致死亡的不良事件(adverse events, AEs)较单纯化疗组少。此外，Durvalumab联合EP组的中位OS长达13个月，有望进一步提高SCLC患者的总生存^[25]^。

### 3.2 维持治疗

既往研究^[19, 20]^表明化疗可以增强肿瘤对免疫治疗的敏感性，而且维持期的患者较疾病进展时具有更好的功能状态。因此，在维持期应用免疫治疗也许更安全有效。

#### 3.2.1 免疫单药

2018年7月公布了一项有关Pembrolizumab在ES-SCLC维持治疗中作用的单臂Ⅱ期研究的数据^[26]^。共纳入了45例广泛期SCLC患者，在他们接受一线铂-依托泊苷化疗后，每3周进行一次Pembrolizumab 200 mg静脉内维持治疗。初步结果提示中位PFS只有1.4个月，没有达到预期的主要终点(3个月)。但是，肿瘤基质细胞程序性死亡配体-1(programmed death ligand-1, PD-L1)表达阳性的患者可以有长期生存获益，中位OS可达12.8个月。

#### 3.2.2 免疫双药联合治疗

随机双盲Ⅲ期试验(CheckMate 451)评估了Nivolumab单独或联合Ipilimumab用于ES-SCLC维持治疗的疗效。834例患者按1:1:1随机分配：单药治疗组每2周接受Nivolumab治疗，联合治疗组在完成4个周期的Nivolumab+Ipilimumab后接受Nivolumab单药维持，其余则接受安慰剂作为对照组。在2019年欧洲肿瘤内科学会(European Society for Medical Oncology, ESMO)会议上公布的结果表明：与安慰剂组相比，联合治疗组与单药治疗组的OS均没有明显延长，其风险比分别为0.92(95%CI: 0.75-1.12)和0.84(95%CI: 0.69-1.02)^[27]^。

总之，目前已有结果的探索维持治疗的试验均没有达到主要终点，但是PD-L1表达阳性的患者似乎可以从pembrolizumab维持治疗中获益。大型临床试验CheckMate 451中具有特定标志物的SCLC患者是否也可以获益仍然值得进一步探讨。未来的试验可在具有特定生物标志物的人群中探讨ICIs在维持治疗中的疗效。

### 3.3 二线及以上治疗

#### 3.3.1 免疫单药

CheckMate032试验评估了在病情进展的SCLC患者中应用Nivolumab或Nivolumab联合Ipilimumab治疗的安全性和有效性。起初进行的是非随机队列研究，共纳入216例既往接受过治疗的患者，接受单药Nivolumab(每2周，3 mg/kg)，或不同组合的Nivolumab+Ipilimumab治疗(1 mg/kg+1 mg/kg，1 mg/kg+3 mg/kg或3 mg/kg+1 mg/kg)。结果表明，单独使用Nivolumab、Nivo1+Ipi3和Nivo3+Ipi1的客观缓解率(objective response rate, ORR)分别为10%、23%和19%，显示出良好的抗肿瘤活性^[28]^。随后进行的随机队列研究表明，Nivolumab联合Ipilimumab组的主要终点ORR较Nivolumab单药组显著提高，分别为21.9%和11.6%。但是联合治疗组在长期总生存方面的获益不如单药治疗组：两组的中位OS分别为4.7个月和5.7个月。并且，Nivolumab联合Ipilimumab组AEs的发生率相对更高(37.5% *vs* 12.9%)，但这些毒性都是可接受的^[29]^。

CheckMate 331试验(一项Ⅲ期临床随机对照研究)比较了Nivolumab单药(*n*=284)与化疗(*n*=285)用于SCLC患者二线治疗中的疗效。研究共纳入569例在一线化疗后复发/进展的SCLC患者，按1:1的比例随机接受Nivolumab或化疗(拓扑替康或氨柔比星)。2018年ESMO会议上公布了该研究的初步结果，发现Nivolumab组与化疗组两组间的OS没有统计学差异(HR=0.86; 95%CI: 0.72-1.04; *P*=0.11)，研究未达到主要观察终点。值得注意的是，在铂耐药SCLC人群中，接受Nivolumab的患者可能有长期生存获益(HR=0.71; 95%CI: 0.54-0.94)^[30]^。

KEYNOTE-028试验的SCLC队列探讨了Pembrolizumab用于二线及以上治疗的临床活性和安全性。该队列共招募了24例PD-L1表达阳性(TPS ≥1%)的广泛期SCLC患者。结果表明，所有患者的客观反应率为33%，优于拓扑替康(标准二线治疗)的9%-23%^[7]^。此外，患者的中位反应持续时间和中位OS分别为19.4个月和9.7个月，展现出临床上有意义的抗肿瘤活性，并且总体耐受性良好^[31]^。随后，KEYNOTE-158的SCLC队列纳入了更多的人群(107例患者)来探索pembrolizumab对复发SCLC患者的疗效。中期数据表明，研究的主要终点ORR为18.7%，中位OS为9.1个月，证实了单药Pembrolizumab是SCLC复发患者的一种有效治疗选择^[32]^。最近的一项研究^[33]^对这两项临床试验的结果进行合并分析，发现纳入人群中共有83例患者既往接受过二线及以上的治疗，他们的ORR为19.3%，其中约61%的应答者反应持续时间超过18个月。基于这些数据，FDA已经批准Pembrolizumab用于在含铂化疗及至少另一种先前的治疗方案后病情进展的晚期SCLC患者。

类似地，一些临床试验也对抗PD-L1抑制剂在二线治疗中的疗效进行了评估。一项涉及17例复发ES-SCLC患者的Ia期研究发现Atezolizumab具有可耐受的安全性，在疗效和生存方面也取得了令人鼓舞的结果，根据免疫相关反应标准确定的ORR为24%^[34]^。但令人遗憾的是，随后的IFCT-1603试验没有达到预期的主要终点。2019年5月公布的数据表明，Atezolizumab组和化疗组(拓扑替康或EP重新诱导化疗)在6周时的ORR分别为2.3%和10%。此外，PFS数据也相当令人失望，Atezolizumab组中位PFS明显比化疗组低(1.4个月*vs* 4.3个月)^[35]^。

此外，有关Durvalumab在SCLC二线及以上治疗的研究表现出良好的安全性。共21例ES-SCLC患者在疾病进展后每2周接受Durvalumab 10 mg/kg治疗，结果显示该方案的毒副作用小，发生的所有免疫相关不良事件均为1级或2级^[36]^。

#### 3.3.2 免疫联合化疗

一项Ⅱ期小队列研究探讨了化疗联合抗程序性死亡受体1(programmed death 1, PD-1)药物对难治性ES-SCLC患者的疗效。共26例接受EP化疗后疾病进展的患者被纳入研究，接受6个周期的紫杉醇治疗，并从第二周期开始联用Pembrolizumab。所有患者的ORR为23.1%，中位OS可达9.2个月。该联合治疗方案的毒性是可以接受的，主要不良事件考虑是由化疗引起的。研究还发现中位PFS与PD-L1表达之间无明显关联(*P*=0.897)。未来需要进一步的研究以确定可能受益于Pembrolizumab联合治疗的患者^[37]^。

#### 3.3.3 免疫双药联合治疗

2018年ASCO会议上报告了PD-L1单抗Durvalumab联合抗CTLA-4单抗Tremelimumab二线治疗ES-SCLC的安全性和有效性。这项Ⅰ期单臂研究中所有患者(*n*=30)的ORR为13.3%，中位反应持续时间可达18.9个月。并且，研究人员观察到在铂敏感和铂耐药/难治性患者中联合治疗的反应均是持久的^[38]^。

BALTIC试验也探讨了Durvalumab联合Tremelimumab应用于铂难治/耐药ES-SCLC的初步疗效。21例患者每4周接受Durvalumab联合Tremelimumab治疗，持续4个月，然后从第16周起单独用Durvalumab治疗。截止至2018年2月2日，中位治疗时间约为14周，ORR为9.5%(95%CI: 1.17%-30.38%)，12周的疾病控制率(disease control rate, DCR)为38.1%，研究中与治疗有关的AEs的发生率约为19%。可见，Durvalumab联合Tremelimumab在难治性复发ES-SCLC中显示出可耐受的安全性，并可以促进抗肿瘤活性^[39]^。

#### 3.3.4 免疫联合放疗

放射治疗可以诱导免疫原性细胞死亡、清除耐药细胞，阻止其扩散到胸腔外^[40, 41]^，因此免疫治疗联合放疗可能是一种有效的治疗方案。一项Ⅱ期随机对照研究评估了免疫双联治疗(Tremelimumab+Durvalumab)联合或不联合立体定向放疗治疗复发性SCLC的疗效。研究共纳入17例患者，其中A组有8例患者，每4周接受Tremelimumab(1, 500 mg)和Durvalumab(75 mg)治疗，B组有9例患者，除了接受Tremelimumab和Durvalumab治疗外，还接受了放疗。2019年在ASCO会议上的初步结果表明，两组之间的疗效没有显著差异，但接受放疗联合免疫双联治疗的中位PFS和OS均有改善的趋势^[42]^。这是目前唯一一项已经有结果的免疫联合放疗治疗SCLC的临床研究，其余的临床试验正在进行中，如表 2所示。

**表 2:** 正在进行的免疫治疗联合放疗应用于小细胞肺癌的临床试验 Ongoing clinical trials of immunotherapy combined with radiotherapy in SCLC

Study ID	Phase	Patient, estimated	Estimated study completion date	Arms	Primary endpoint
NCT03811002	II/III	506	December 28, 2026	Etoposide+Cisplatin/carboplatin+RT (LS-SCLC)±Atezolizumab	PFS, OS
NCT02934503	II	60	October 2020	Cisplatin/carboplatin+Etoposide+ Pembrolizumab±RT	Change in PD-L1 expression status
NCT02402920	I	80	July 31, 2023	Cisplatin/carboplatin+Etoposide+ Pembrolizumab+RT	AEs
NCT03540420	II	212	December 2026	Chemo-radiotherapy±Atezolizumab	2-year survival
NCT03262454	II	35	July 31, 2024	Atezolizumab±RT	ORR
NCT03043599	I/II	21	April 2022	Ipilimumab+Nivolumab+TRT	Phase Ⅰ: Confirmation of recommended phase Ⅱ dose; Phase Ⅱ:PFS
NCT03585998	II	51	December 19, 2021	Radiotherapy+Etoposide+Cisplatin+Durvalumab, followed by consolidationdurvalumab	PFS
NCT03923270	NA	54	May 1, 2025	TRT+Durvalumab±Tremelimumab/Olaparib	AE, PFS
NCT03509012	I	360 (Advanced solidtumors)	April 4, 2022	Durvalumab+Cisplatin/carboplatin+Etoposide+RT	DLTs, AEs
TRT: thoracic radiotherapy; RT: radiation therapy; DLTs: dose limiting toxicities; AEs: adverse events.					

## 4 预测标志物

近年来，免疫微环境在SCLC中的作用逐渐引起关注，识别SCLC肿瘤微环境中的生物标志物有助于预测患者的生存。与肿瘤微环境相关的基因*GZMB*、*HAVCR2*、*PRF1*和*TBX2*可以作为监测肿瘤对ICIs反应的标志物^[43]^。Muppa等^[44]^发现在长期存活的SCLC中，肿瘤浸润和相关淋巴细胞数量增加，患者的生存受到肿瘤微环境中免疫细胞的影响。因此，增强抗肿瘤免疫反应的个性化免疫治疗策略可能在SCLC的治疗中具有广阔的前景。一项涉及104例SCLC的回顾性分析表明，Ⅰ期-Ⅲ期SCLC中PD-L1阳性表达显著高于转移患者，FOXP3+肿瘤浸润淋巴细胞(tumour-infiltrating lymphocytes, TILs)的存在是较早期(Ⅰ期-Ⅲ期)SCLC的潜在预后标志物^[45]^。也有研究^[46]^发现肿瘤细胞或TILs中PD-L1的高表达与生存期缩短有关，而CD3、CD20和CD45的高表达与生存期延长有关。此外，在伴有神经性副肿瘤综合征的SCLC肿瘤组织中，TILs、PD-L1表达和PD-1/PD-L1相互作用增加，提示具有神经性副肿瘤综合征的SCLC患者具有炎症性肿瘤微环境，可能是免疫治疗的理想人群^[47]^。

一些SCLC免疫治疗的临床试验陆续公布了对生物标志物的探索结果。Arriola等^[23]^发现，自身抗体阳性表达与Ipilimumab联合化疗的治疗反应之间存在一定的相关性(*P*=0.066)。抗核抗体阳性预示着免疫相关PFS显著延长(10.2个月*vs* 6.9个月，*P*=0.032)，并且自身免疫性抗体阳性的患者倾向于有更好的生存(18.5个月*vs* 17个月，*P*=0.144)。但是，由于缺乏对照组且既往有研究表明基线时自身抗体的存在与SCLC患者的预后相关^[14, 48]^，该研究无法证明自身抗体阳性是具有预测疗效的作用还是仅仅具有预后价值。

SCLC中PD-L1的表达频率相对较低。CheckMate-032研究中PD-L1表达≥1%的患者约为17%，而表达≥5%的患者更少，仅有5%。探索性分析发现，Nivolumab单药治疗或与Ipilimumab联合治疗的临床疗效与PD-L1表达无关^[28]^。但是，KEYNOTE-028^[31]^和KEYNOTE-158^[32]^试验发现PD-L1在基质细胞中表达似乎更频繁，可达到39%，并且和患者的生存结果显著相关。在KEYNOTE-158试验中，肿瘤PD-L1表达阳性的患者生存优势更明显：ORR超过35%，中位OS可达到14.6个月。造成这些试验结果差异的原因可能是PD-L1表达的评估方式不同。在有关Nivolumab的研究中，PD-L1阳性定义为在包括至少100个可评估的肿瘤细胞的样本中肿瘤细胞PD-L1≥1%。而在有关Pembrolizumab的试验中，至少包括50个可评估的肿瘤细胞即为合格的肿瘤组织样本，PD-L1的表达不仅存在于肿瘤细胞中，也存在于基质细胞(比如与肿瘤相关的巨噬细胞或淋巴细胞)。这意味着，在KEYNOTE试验中合格的组织样本也许在CheckMate试验中是不可评估的，而且KEYNOTE试验中PD-L1阳性是基于在肿瘤和周围微环境中的综合评分。在将来的研究中，有必要统一PD-L1的评估方式。

TMB是另一种对ICIs敏感的标志物^[49]^。CheckMate 032试验中，Hellmann等^[50]^对参加研究的211例患者配对的肿瘤组织和全血样本进行了全外显子组测序，并基于错义突变总数将TMB分为低TMB(0个-143个突变)、中TMB(143个-247个突变)和高TMB(≥248个突变)三组。研究发现，免疫单药组高、中、低TMB患者的ORR分别为21.3%、6.8%和4.8%，而在免疫联合治疗组中则分别为46.2%、16.0%和22.2%。与具有中等或低TMB的患者相比，具有高TMB的患者接受Nivolumab单药治疗或免疫双联治疗的疗效更好。此外，在未接受免疫治疗的SCLC患者的独立队列中，患者的总生存与TMB无关。这些数据表明TMB可以作为Nivolumab单独或联合Ipilimumab治疗的有效预测因子。

由于不是所有的患者都可以获得足够的肿瘤组织样本，人们越来越关注通过血液循环中的肿瘤细胞DNA来评估TMB。既往已有研究证实了基于血液评估的TMB可以替代肿瘤组织TMB作为接受Atezolizumab治疗的晚期非小细胞肺癌(non-small cell lung cancer, NSCLC)患者的潜在预测标志物^[51]^。同样地，IMpower133试验基于血液样本对TMB进行了评估。令人遗憾的是，研究人员最终没有在初治广泛期SCLC患者中观察到血浆TMB与Atezolizumab联合卡铂/依托泊苷治疗疗效之间的相关性^[24]^。

总之，SCLC大多为免疫沙漠表型，具有高肿瘤突变负荷，探讨TMB的最佳阈值有助于预测ICIs的疗效; 也有少部分为炎症性肿瘤，这部分肿瘤高表达PD-L1，含有较多浸润肿瘤的免疫细胞或淋巴细胞，此时，PD-L1也许是有效的预测指标^[52, 53]^。因此，PD-L1表达联合TMB联合也许具有更有效的预测能力。但是，由于肿瘤微环境的组成随时间和肿瘤进展阶段的不同而变化^[52]^，不同肿瘤部位的组织(原发病灶*vs*转移灶)、样本类型(手术切除*vs*活检)及采样时间(档案组织*vs*新鲜的预处理组织)可能会影响标志物的表达，选择合适的肿瘤部位、样本类型以及采样时间需要更深入的研究。

## 5 总结与展望

免疫治疗在SCLC治疗领域取得了重大的突破，尤其为一线和三线治疗提供了更多的选择。但是，其生存获益仅限于一小部分人群，这提示需要可靠的预测性标志物来探索优势人群。

就已有结果而言，PD-L1的预测作用只限于Pembrolizumab小样本研究且仅在基质细胞中表达阳性时有效。另一方面，肿瘤突变负荷总体上与ICIs疗效相关，特别是Nivolumab单独或联合Ipilimumab治疗。但是TMB的最佳截止值尚无统一标准且目前相关临床研究较少，不具有普遍性。此外，与NSCLC不同，血浆TMB似乎与SCLC免疫治疗疗效无关。因此，组织学TMB联合基质细胞PD-L1表达可能是目前最有预测价值的标志物。在今后的研究中，有必要设计早期临床试验，用不同的分子标记物做亚组来入组患者以探讨其预测价值。

总之，除了将ICIs单独应用或与化疗联合应用之外，未来还需要继续探索新的治疗方案(如免疫联合放疗或免疫双联治疗等)和其他预测性生物标记物，使患者获得最优化治疗。
